# Forty-year prognosis after plaque brachytherapy of uveal melanoma

**DOI:** 10.1038/s41598-020-68232-7

**Published:** 2020-07-09

**Authors:** Gustav Stålhammar

**Affiliations:** 1 St. Erik Eye Hospital, Polhemsgatan 50, 112 82 Stockholm, Sweden; 2 Department of Clinical Neuroscience, Karolinska Institutet, 171 77 Stockholm, Sweden

**Keywords:** Eye cancer, Outcomes research

## Abstract

In this study, the long-term patient survival after plaque brachytherapy of uveal melanoma is examined. All patients treated between 1980 and 1999 at a single institution were included (*n* = 677). 533 (79%) had deceased before the end of follow-up. The median follow-up for the 144 survivors was 25.4 years (SD 5.2). Uveal melanoma-related mortality was 18% by 5 years, 28% by 10 years, 32% by 15 years, 35% by 20 years, and 36% by 25 to 40 years. 172 of 209 (82%) uveal melanoma-related deaths occurred within the first decade after brachytherapy. Relative survival rates were 74% at 5 years, 64% at 10 years, 62% at 20 years, 83% at 30 years and ≥100% at 32 to 40 years. Tumor diameter and local recurrence were independent predictors of uveal melanoma-related mortality in multivariate Cox proportional hazards analysis. In conclusion, uveal melanoma has a high mortality rate and most uveal melanoma-related deaths occur in the first decade after treatment. Long-term survivors may have a survival advantage to individuals of the same sex and age from the general population.

## Introduction

Uveal melanoma is the most common malignant tumor arising in the interior of the eye^1^. At the time of diagnosis, less than 5% of patients have detectable metastases^2^. But even if the tumor eye is immediately removed, up to 50% of patients will develop metastases within 25 years^3^. This has been attributed to early seeding of micrometastases from the eye to distant organs^4^. Once these small clusters of dormant tumor cells start growing into larger and radiologically detectable macrometastases, no treatment has proven effective and median patient survival is less than a year^5,6^.

Most early studies reporting 15-year survival or longer for patients with uveal melanoma exclusively included patients that underwent surgical treatment^3,7–9^. In 2001, the large randomized Collaborative Ocular Melanoma Study (COMS) showed equal 5-year survival rates after enucleation and plaque brachytherapy of tumors with an apical thickness of 2.5 to 10 mm and a basal diameter of less than 15 mm^10^. Later, the 10-year uveal melanoma-related mortality was reported to be 21 and 22% in the enucleation and brachytherapy arms, respectively^11^. Consequently, there is reason to believe that the two types of treatment have equivalent outcomes within this size interval, and that survival after plaque brachytherapy can be regarded as a proxy for survival after enucleation.

Within the Swedish population of currently 10 million, all uveal melanoma patients undergoing plaque brachytherapy are treated at our institution only, permitting collection of extensive patient, treatment and survival data with minimal loss to follow-up. Here, plaque brachytherapy has been the treatment of choice for tumors with an apical height of less than 10 mm for four decades. Eyes with thicker tumors are generally enucleated, whereas eyes with tumors with an apical height of less than 2.5 mm are observed for growth.

In this paper, disease-specific, overall and relative survival as well as hazard for uveal melanoma-related mortality is investigated for all patients treated in our institution between years 1980 through 1999.

## Results

### Descriptive statistics

A total of 677 patients with 677 uveal melanomas originating in the choroid or ciliary body were eligible for analysis. Their mean age at diagnosis was 62 years (standard deviation (SD) 14) with similar proportions of men (46%) and women (54%) as well as right (47%) and left (53%) eyes included. The mean thickness at tumor apex was 5.2 mm (SD 2.5), with a mean diameter of 10.1 mm (SD 3.8). 659 of the 677 tumors (97%) were of T-category 1, 2 or 3 according to the American Joint Committee on Cancer (AJCC) classification. Mean radiation dose was 93.9 Gy (Gy) at the tumor apex (SD 18.5) and 633.6 Gy at the scleral surface (SD 371.6). Median follow-up was 25.4 years (SD 5.2, Table 1).

**Table 1:** Characteristics of patients and tumors included in this study.

***n = ***	677
**Mean age at brachytherapy, years (SD)**	62 (14)
**Sex, n (%)**	
Female	363 (54)
Male	314 (46)
**Tumor eye laterality, n (%)**	
Right	321 (47)
Left	356 (53)
**Mean tumor thickness, mm (SD)**	5.2 (2.5)
**Mean tumor diameter, mm (SD)**	10.1 (3.8)
**Mean tumor distance to optic disc, mm (SD)**	4.0 (3.4)
**AJCC T-category, n (%)**	
1	302 (45)
2	265 (39)
3	92 (14)
4	18 (3)
**AJCC stage, n (%)**	
1	302 (45)
2a	265 (39)
2b	92 (14)
3a	18 (3)
3b	0 (0)
3c	0 (0)
4	0 (0)
**Radiation dose, mean (SD)**	
At tumor apex	93.9 Gy (18.5)
At scleral surface	633.6 Gy (371.6)
**TTT, n (%)**	3 (0.4)
**Median follow-up, years (SD, min–max)**	25.4 years (5.2, 20.4–40.1)

SD, standard deviation; AJCC, American Joint Committee on Cancer; Gy, Gray; TTT, transpupillary thermotherapy given prior to or at the time of brachytherapy.

### Survival

Of the 677 patients included, 533 (79%) had deceased before the end of follow-up. Of these 533 patients, 209 (39%) had deceased from uveal melanoma. Uveal melanoma-related mortality was 18% 5 years after plaque brachytherapy, 28% at 10 years, 32% at 15 years, 35% at 20 years, and 36% at 25, 30, 35 and 40 years, according to cumulative incidence analysis. (Table 2). Of the 209 uveal melanoma-related deaths, 119 (57%) occurred within 5 years from plaque brachytherapy. 172 (82%) occurred within the first decade, and 195 of 209 (93%) within 15 years. Only 3 uveal melanoma-related deaths occurred for 212 patients surviving more than 20 years after brachytherapy, and no uveal melanoma-related deaths occurred for 124 patients surviving more than 25 years. All-cause mortality was 27% at 5 years, 45% at 10 years, 57% at 15 years, 69% at 20 years, 77% at 25 years, 81% at 30 years, 84% at 35 years and 86% at 40 years (Table 2).

**Table 2:** Cumulative uveal melanoma-related and all-cause mortality.

Time after brachytherapy (years)	Uveal melanoma-related mortality (%)	All-cause mortality (%)
1	2	3
5	18	27
10	28	45
15	32	57
20	35	69
25	36	77
30	36	81
35	36	84
40	36	86

### Kaplan–Meier

**Figure 1:**
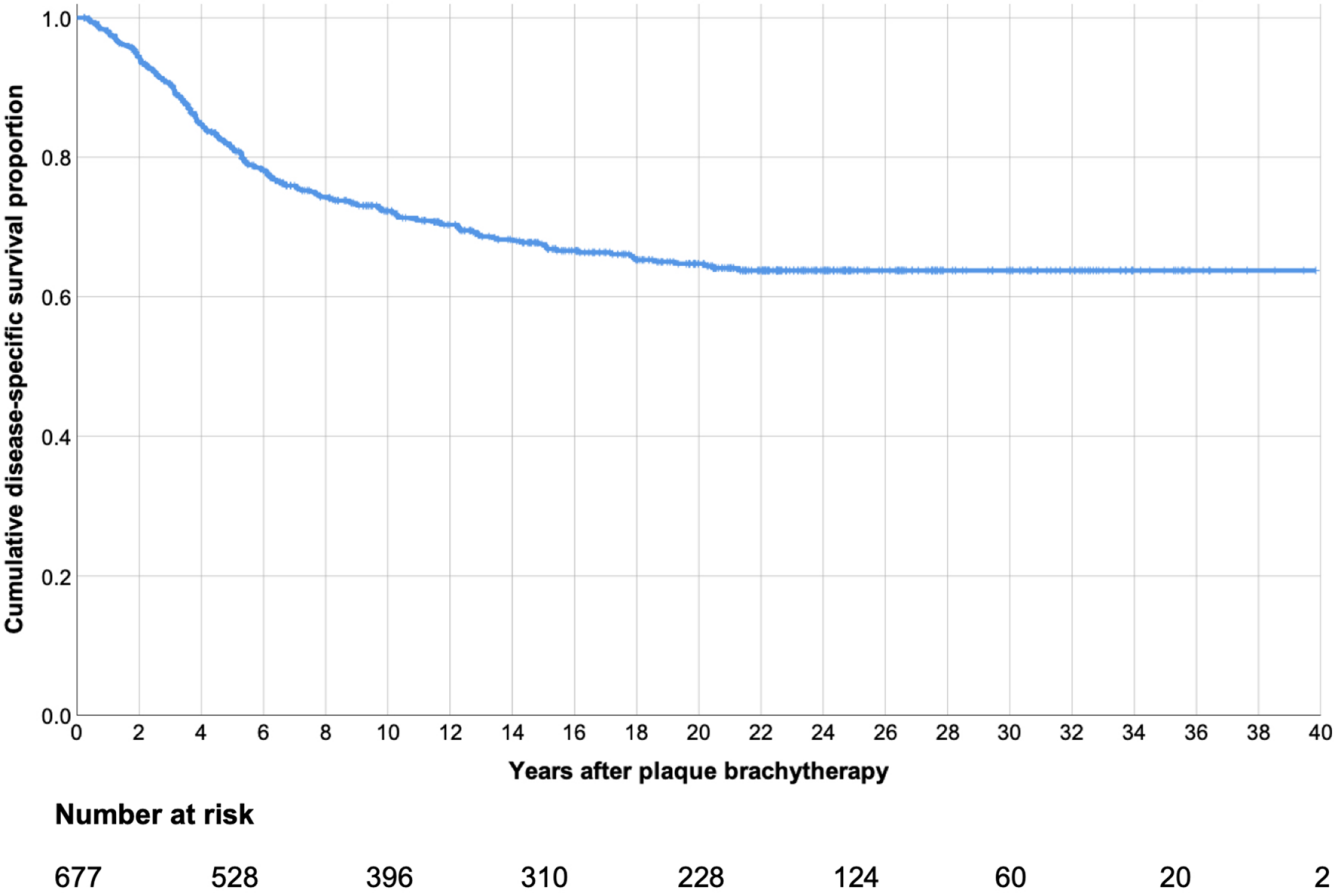
Kaplan–Meier curve, cumulative disease-specific survival proportion after plaque brachytherapy of uveal melanoma.

**Figure 2:**
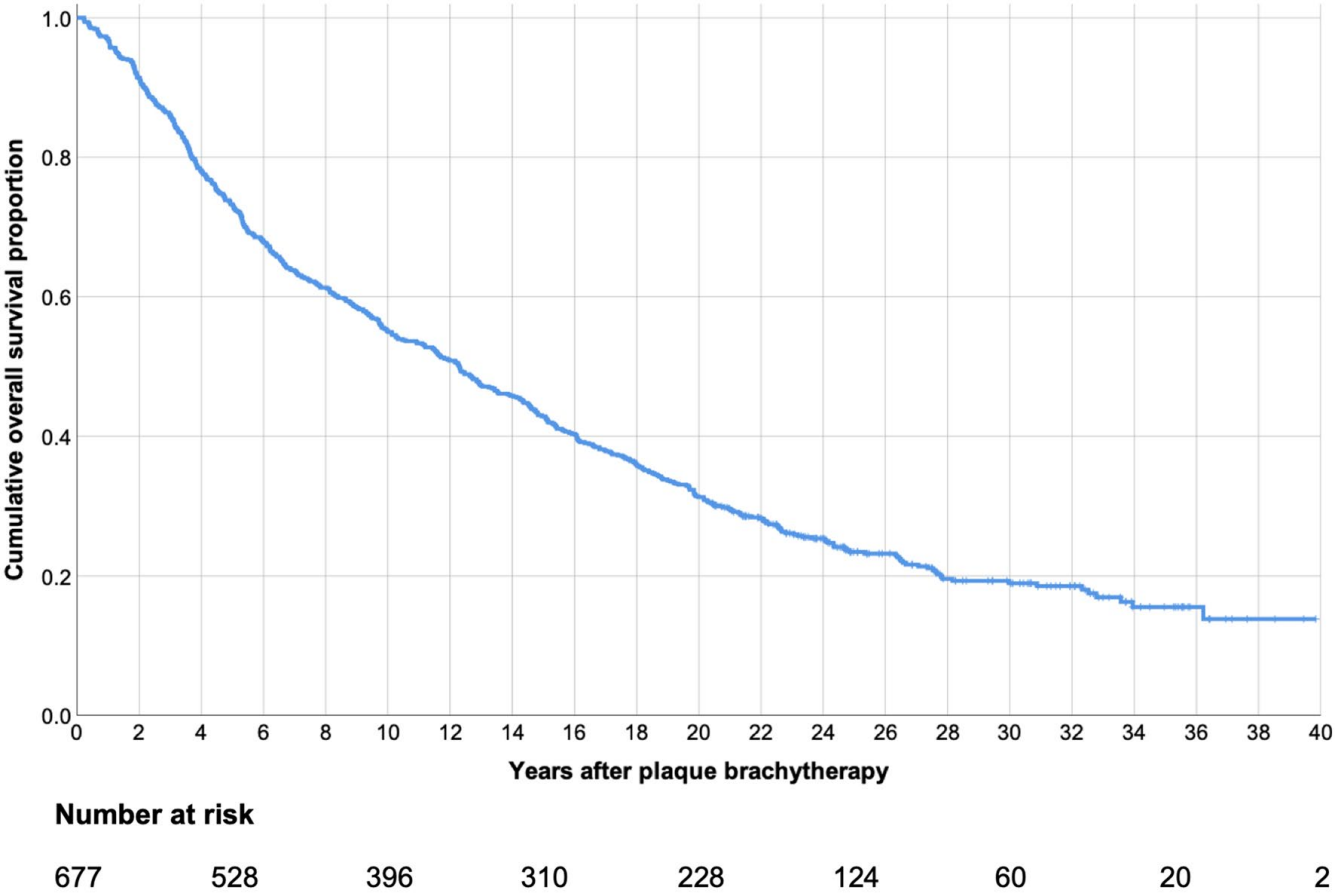
Kaplan–Meier curve, cumulative overall survival proportion after plaque brachytherapy of uveal melanoma.

The disease-specific survival did not meet the median. Mean disease-specific survival was 27.8 years (95% confidence interval (CI) 26.5–29.1, Fig. 1). Kaplan–Meier disease-specific survival was 98% at 1 year, 82% at 5 years, 72% at 10 years, 68% at 15 years, 65% at 20 years and 64% at 25 to 40 years. Median overall survival was 12.3 years (95% CI 10.7–13.9, Fig. 2).

### Relative survival rates

**Figure 3:**
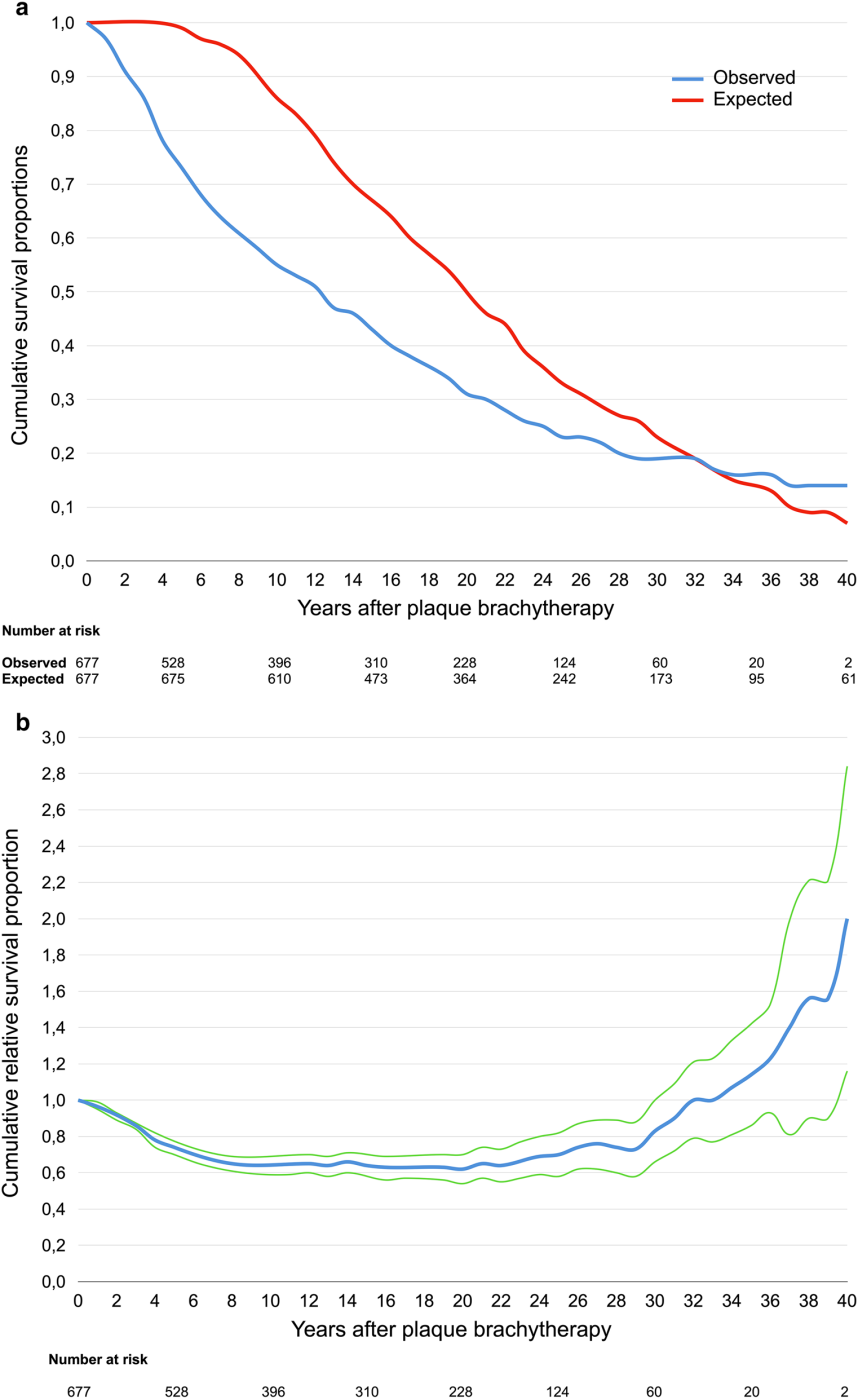
**(a)** Cumulative observed (blue) and expected (red) survival proportions after plaque brachytherapy of uveal melanoma. **(b)** Cumulative relative survival proportion after plaque brachytherapy of uveal melanoma (blue line). Green lines represent limits of 95% confidence interval.

The observed survival proportions of patients with uveal melanoma were 97% at 1 year after plaque brachytherapy. 73% at 5 years after plaque brachytherapy, 55% at 10 years, 43% at 15 years, 31% at 20 years, 23% at 25 years, 19% at 30 years, 16% at 35 years and 14% at 40 years. The expected survival proportions of persons in the general population with the same sex and age at the same calendar year as our patients were 100% at 1 year after plaque brachytherapy, 99% at 5 years, 86% at 10 years, 67% at 15 years, 50% at 20 years, 33% at 25 years, 23% at 30 years, 14% at 35 years and 7% at 40 years (Fig. 3a). Consequently, the relative survival rates were 97% at 1 year, 74% at 5 years, 64% at 10 years, 64% at 15 years, 62% at 20 years, 70% at 25 years, 83% at 30 years, 114% at 35 years and 200% at 40 years (Fig. 3b, Table 3).

**Table 3:** Cumulative observed, expected and relative survival.

Year after brachytherapy	Observed survival proportion	Expected survival proportion	Relative survival proportion	SE of observed survival rate	95% CI lower limit	95% CI upper limit
1	0.97	1.00	0.97	0.01	0.95	0.99
2	0.91	1.00	0.91	0.01	0.89	0.93
3	0.86	1.00	0.86	0.01	0.84	0.88
4	0.78	1.00	0.78	0.02	0.74	0.82
5	0.73	0.99	0.74	0.02	0.70	0.78
6	0.68	0.97	0.70	0.02	0.66	0.74
7	0.64	0.96	0.67	0.02	0.63	0.71
8	0.61	0.94	0.65	0.02	0.61	0.69
9	0.58	0.90	0.64	0.02	0.60	0.69
10	0.55	0.86	0.64	0.02	0.59	0.69
11	0.53	0.83	0.64	0.02	0.59	0.69
12	0.51	0.79	0.65	0.02	0.60	0.70
13	0.47	0.74	0.64	0.02	0.58	0.69
14	0.46	0.70	0.66	0.02	0.60	0.71
15	0.43	0.67	0.64	0.02	0.58	0.70
16	0.40	0.64	0.63	0.02	0.56	0.69
17	0.38	0.60	0.63	0.02	0.57	0.70
18	0.36	0.57	0.63	0.02	0.56	0.70
19	0.34	0.54	0.63	0.02	0.56	0.70
20	0.31	0.50	0.62	0.02	0.54	0.70
21	0.30	0.46	0.65	0.02	0.57	0.74
22	0.28	0.44	0.64	0.02	0.55	0.73
23	0.26	0.39	0.67	0.02	0.57	0.77
24	0.25	0.36	0.69	0.02	0.59	0.80
25	0.23	0.33	0.70	0.02	0.58	0.82
26	0.23	0.31	0.74	0.02	0.62	0.87
27	0.22	0.29	0.76	0.02	0.62	0.89
28	0.20	0.27	0.74	0.02	0.60	0.89
29	0.19	0.26	0.73	0.02	0.58	0.88
30	0.19	0.23	0.83	0.02	0.66	1.00
31	0.19	0.21	0.90	0.02	0.72	1.09
32	0.19	0.19	1.00	0.02	0.79	1.21
33	0.17	0.17	1.00	0.02	0.77	1.23
34	0.16	0.15	1.07	0.02	0.81	1.33
35	0.16	0.14	1.14	0.02	0.86	1.42
36	0.16	0.13	1.23	0.02	0.93	1.53
37	0.14	0.10	1.40	0.03	0.81	1.99
38	0.14	0.09	1.56	0.03	0.90	2.21
39	0.14	0.09	1.56	0.03	0.90	2.21
40	0.14	0.07	2.00	0.03	1.16	2.84

*SE* standard error, *CI* confidence interval.

### Long-term survivors

The 209 of our 677 patients that succumbed to their disease were of a similar age at diagnosis as the 468 patients that survived or died from other causes (62 vs. 61 years, Student’s t-test *p* = 0.468) and had tumors at similar distance to the optic disc (*p* = 0.494), but they had significantly larger tumors (diameter *p* < 0.001, thickness *p* < 0.001, AJCC T-category *p* < 0.001) at significantly more advanced anatomic stage (AJCC stage *p* < 0.001).

Out of the originally 677 patients, 373 (55%) survived 10 years or longer after plaque brachytherapy. These patients were significantly younger at diagnosis (Student’s t-test *p* < 0.001) and had tumors with significantly smaller diameters (*p* < 0.001), thinner apical thickness (*p* < 0.001) and lower AJCC stage (*p* < 0.001) than patients that died within 10 years. Tumor distance to the optic disc was however not significantly different between the groups (*p* = 0.566, Table 4a).

**Table 4:** Mean patient and tumor characteristics for patients surviving shorter vs. longer than (a) 10 years, (b) 20 years, (c) mean patient and tumor characteristics for patients with shorter vs. longer than 30 years of follow-up.

	Patients surviving < 10 years after brachytherapy	Patients surviving ≥ 10 years after brachytherapy	*p*
**(a)**			
Mean patient age at diagnosis, years	66.3	57.7	< 0.001
Mean tumor diameter, mm	11.5	9.2	< 0.001
Mean tumor thickness, mm	5.8	4.9	< 0.001
Mean AJCC stage 1–4	2.0	1.6	< 0.001
Mean distance to optic disc, mm	4.1	3.9	0.552

	Patients surviving < 20 years after brachytherapy	Patients surviving ≥ 20 years after brachytherapy	*p*
**(b)**			
Mean patient age at diagnosis, years	65.6	52.5	< 0.001
Mean tumor diameter, mm	10.7	8.9	< 0.001
Mean tumor thickness, mm	5.4	4.9	0.769
Mean AJCC stage 1–4	1.9	1.5	< 0.001
Mean distance to optic disc, mm	4.2	3.7	0.131

	Patients with < 30-year follow-up	Patients with ≥ 30-year follow-up	*p*
**(c)**			
Mean patient age at diagnosis, years	62.9	46.1	< 0.001
Mean tumor diameter, mm	10.2	9.0	< 0.001
Mean tumor thickness, mm	5.2	5.3	0.033
Mean AJCC stage 1–4	1.8	1.6	0.197
Mean distance to optic disc, mm	4.1	3.4	0.208

212 patients (31%) survived 20 years or longer. Similarly, these were significantly younger at diagnosis (*p* < 0.001) and had tumors with significantly smaller diameters (*p* < 0.001), thinner apical thickness (*p* = 0.033) and lower AJCC stage (*p* < 0.001). Tumor distance to the optic disc was not significantly different between the groups (*p* = 0.131, Table 4b).

54 patients (8%) had 30 years of follow-up or longer. The remaining 623 were either dead or had shorter follow-up. In contrast to the 10 and 20-year survivor groups, the only significant differences for these patients were the age at diagnosis (*p* < 0.001) and tumor diameter (*p* < 0.019). Tumor apical thickness, AJCC stage and distance to the optic disc were not significantly different to patients with shorter follow-up (Table 4c).

In binary logistic regression with patient age at diagnosis, tumor diameter, tumor thickness, AJCC stage, distance to the optic disc and local recurrence as covariates, patient age at diagnosis (odds ratio 0.96 per increased year, *p* < 0.001) and tumor diameter (odds ratio 0.84 per increased mm, *p* = 0.004) but none of the other variables were independent predictors of survival 10 years or longer after plaque brachytherapy. Similarly, patient age at diagnosis (odds ratio 0.92 per increased year, *p* < 0.001) and tumor diameter (odds ratio 0.86 per increased mm, *p* = 0.017) were independent predictors of survival 20 years or longer. Only patient age at diagnosis (odds ratio 0.91 per increased year, *p* < 0.001) was an independent predictor of survival 30 years or longer after plaque brachytherapy.

### Cox regression

**Figure 4:**
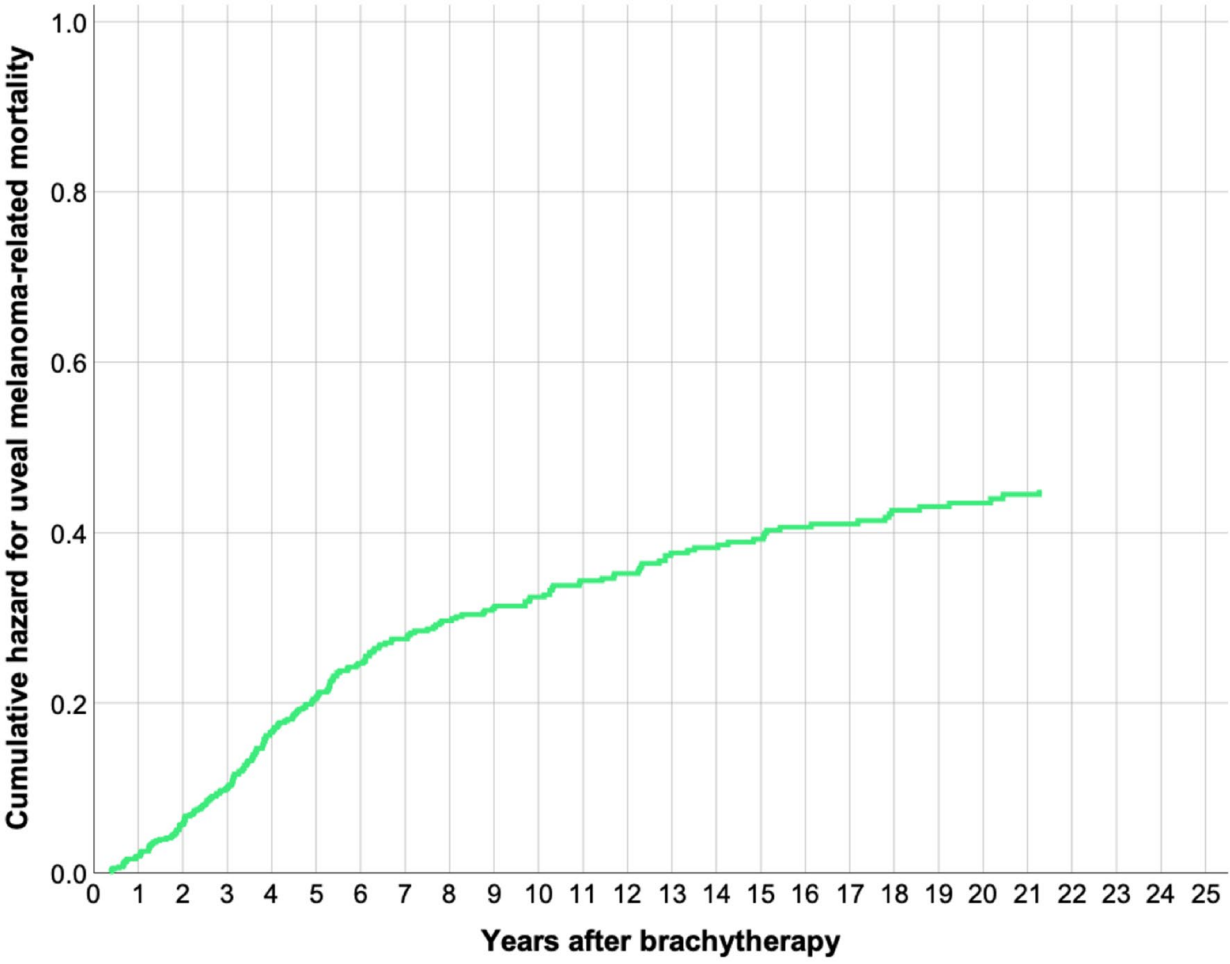
Cox regression cumulative hazard for melanoma-related mortality (0.21 at 5 years, 0.32 at 10 years, 0.39 at 15 years and 0.44 at 20 years).

The Cox regression hazard for uveal melanoma-related mortality was 0.21 at 5 years, 0.32 at 10 years, 0.39 at 15 years and 0.44 at 20 years (Fig. 4). In multivariate analysis with patient age at diagnosis, tumor diameter, tumor thickness, AJCC stage, distance to the optic disc and local recurrence as covariates, tumor diameter (hazard ratio 1.1 per increased mm, 95% CI 1.0–1.3, *p* = 0.006) and local recurrence (hazard ratio 1.1, 95% CI 1.1–2.4, *p* = 0.015), were independent predictors of uveal melanoma-related mortality (Table 5).

**Table 5:** Multivariate Cox proportional hazards analysis.

Covariate	Regression coefficient, β (SE)	Wald statistic	*p*	Hazard coefficient, Exp(b) (95% CI)
Patient age at diagnosis, years	0.002 (0.007)	0.1	0.746	1.0 (1.0–1.0)
Tumor diameter, mm	0.1 (0.1)	7.6	0.006	1.1 (1.0–1.3)
Tumor thickness, mm	0.1 (0.06)	2.2	0.134	1.0 (1.0–1.2)
AJCC stage 1–4	− 0.03 (0.3)	0.02	0.895	1.0 (0.6–1.6)
Distance to optic disc, mm	− 0.0004 (0.03)	0.0003	0.985	1.0 (0.9–1.1)
Local recurrence, yes/no	0.5 (0.2)	5.9	0.015	1.1 (1.1–2.4)

## Discussion

In this study, the long-term survival after plaque brachytherapy of uveal melanoma has been examined. The uveal melanoma-related mortality reported herein is higher than what was found in the brachytherapy arm of the COMS, in which the 5, 10 and 12 year uveal melanoma-related mortality rates were 10%, 18%, and 21%, respectively^11^. On the other hand, Kujala et al. reported mortality rates as high as 31%, 45% and 49% at 5, 10 and 15 years^3^. Other than differences in methodology, the results in the latter study may at least partially be explained by a larger mean tumor size.

The vast majority of our patients died within 10 years from primary tumor treatment and uveal melanoma-related deaths after 20 years were a rare exception. As suggested by our relative survival rates, patients surviving 20 years after brachytherapy are at no survival disadvantage and even appear to have a survival *advantage* to the general population. Similar time-dependent decreasing survival differences between samples of patients with other aggressive diseases and the general population have been reported before. Data on conditional relative survival rates from the Surveillance, Epidemiology, and End Results (SEER) program by the National Cancer Institute indicate that American patients with breast cancer, colorectal cancer and Hodgkin’s disease catch up some of their excess mortality during the first year after treatment and that their 5-year relative survival gradually increases if counting from 1, 2 or 3 years after diagnosis^12^. In 2014, Davis et al. showed increased mortality rates of patients treated for severe sepsis until 2 years post admission, after which it plateaued and even dropped behind the mortality rate of the general population^13^.

It is unlikely that the increasing relative survival was caused by failure to register deaths on our part. In Sweden, death certificates are legally required for burial, making the data on the number of deaths used for calculation of relative survival complete. Further, 96% of individuals in the Cause of Death Register have a specific underlying cause of death recorded^14^. The similarity of our 1 minus relative survival and the uveal melanoma-related mortality indicates that the data have indeed included a very high proportion of the uveal melanoma-related deaths. Perhaps the increasing relative survival could at least partially be explained by an increased presence of other risk factors for death among those dying from uveal melanoma, and a reduced presence of such factors among the survivors. Positive health benefits from regular follow-ups, increased watchfulness, lowered threshold for intervention and medication, and increased health awareness among those having survived severe disease may also have influenced this outcome.

Few individuals remained after 35 years in both our patient sample and the reference population, which necessitates caution in the interpretation of late events and prevents far-reaching conclusions.

Another limitation to this study is our limited control over confounding factors. Other diseases and risk factors for early death was not recorded and we do not know if their distribution in our patient sample were actually different from the general population. Third, the degree of tumor pigmentation, which has been associated with both prognosis^15,16^ and therapeutic response^17–19^, was not recorded in our data. Fourth, our patients were sampled from one institution only which may limit the generalizability of our findings.

In conclusion, uveal melanoma is associated with a high mortality rate even at relatively small primary tumor sizes. Most uveal melanoma-related deaths occur within 10 years from treatment. Patients surviving 20 years or more after treatment are at no survival disadvantage and may even have a survival advantage over the general population. We encourage future research to clarify if this phenomenon can be confirmed in cohorts with longer follow-up.

## Methods

### Patients and samples

The collection of patients and data for this study was analogous with previous retrospective studies of patients that have undergone plaque brachytherapy at our institution^16,17^.

This study adhered to the tenets of the Declaration of Helsinki and was approved by the regional ethical review board in Stockholm (reference number 2016/247-31/4; and amendment 2019-03485) which also waived the informed consent as this is a retrospective chart review that did not affect the treatment or follow-up of patients. The research was performed in accordance with relevant guidelines. All patients who received plaque brachytherapy for choroidal or ciliary body melanoma at St. Erik Eye Hospital from January 1980 through December 1999 were considered for the study (*n* = 732). Retrospective data was retrieved from our digital Brachytherapy of Uveal Melanoma directory, including information on gender, age at diagnosis, presenting symptoms, symptom duration before presentation, tumor thickness and diameter, AJCC T-category, stage as well as dates of diagnosis and last follow-up. The directory is continuously updated with dates of death from the Swedish population register. As death certificates are legally required for burial, the data is complete save for emigrants. In review of the data, 55 patients were excluded because of incomplete plaque brachytherapy treatment data, yielding a total of 677 patients eligible for analysis.

### Brachytherapy

Ruthenium-106 was the only radioisotope in use for plaque brachytherapy during the study period (CIB, CIA, COB, CCA, CCB, CCX or CCZ types, Eckert & Ziegler BEBIG GmbH, Berlin, Germany). Generally, brachytherapy was the preferred treatment alternative for tumors with a thickness of < 7 mm. In cases where the tumor eye was the patient’s last seeing eye or where the patient strongly preferred eye-preserving therapy, thicker tumors could be considered for plaque brachytherapy as long as the scleral dose would not exceed 1,500 Gy. According to our protocol, a dose of 100 Gy was prescribed at the tumor apex, with 1 mm added to adjust for the typical distance between the plaque and the tumor base. Starting in the year 2000, Iodine-125 plaques were introduced as the first option for tumors with an apical height of 6 to 10 mm. Previous studies have shown that ocular outcomes and patient survival are similar after Ruthenium-106 and Iodine-125 plaque brachytherapy of both thinner and thicker tumors^20,21^. Standardized A- and B-scan ultrasonography was used preoperatively to measure tumor thickness and diameter. Surgery was performed under general anesthesia and included transillumination to identify the tumor’s position, followed by plaque positioning with a minimum of 2-mm safety margin around the tumor. As the diameter of the largest plaques is 20 mm, the maximum diameter of tumors treated with plaque brachytherapy is thereby 16 mm. Tumors close to or immediately under the fovea had eccentric plaque fixation and adjunctive transpupillary thermotherapy to compensate for the compromised radiotherapy margin. Juxtapapillary melanomas were either treated with a notched plaque or a standard plaque combined with adjunctive transpupillary thermotherapy.

### Follow-up

Routine follow-up was scheduled at 1, 3, 6, and 12 months after brachytherapy. If local control had been achieved, patients were then seen annually or semi-annually for the rest of their lives. After a few years, patients living outside the Stockholm area could be referred for follow-ups at their home clinic, with re-referral if tumor growth or complications were discovered. At each visit, best-corrected visual acuity and intraocular pressures were measured. The tumor eye was examined with slit lamp biomicroscopy, ultrasonography, and fundus photography. If tumor regression was insufficient or the tumor progressed 6 months after brachytherapy or at any later point, another round of brachytherapy or enucleation could be considered. At diagnosis, patients underwent radiological screening for metastases with a chest x-ray and an ultrasonography of the liver, or by computed tomography (CT) of the chest and abdomen. The metastasis screening of the liver was then repeated by ultrasonography or by CT semi-annually for 5 years after diagnosis.

### Relative survival

To calculate relative survival rates, we used generalized data from the Swedish life tables with year-per-year statistics for persons from the general population^22^. Each patient was assigned a remaining life expectancy based on his or her sex, age at the time of uveal melanoma diagnosis and the calendar year at diagnosis. For example, a 60-year old man in the general population had a remaining life expectancy of 17.9 years in the first year of the study (1980), but 20.4 in the last year of the study (1999, Supplementary Table 1). A 60-year old woman had a remaining life expectancy of 22.1 and 24.2 years during the same years, respectively (Supplementary Table 2). This remaining life expectancy was then compared with the observed survival to calculate relative survival rates.

### Statistical analysis

Differences with a *p* < 0.05 were considered significant, all *p* values being 2-sided. When evaluated by the Shapiro–Wilk test, the deviation from normal distribution was not statistically significant for any of our continuous variables (*p* > 0.05) and all variances were equal (Levene's test for equality of variances *p* > 0.05). We therefore used independent samples Student's t-test with equal variances assumed when comparing continuous variables between groups. For evaluation of the association between clinicopathological variables and long-term survivorship, we used binary logistic regression with multiple covariates. For analysis of outcome, Cox Regression Hazards for uveal melanoma-related mortality, Kaplan–Meier disease-specific survival, Kaplan–Meier overall survival as well as relative survival were calculated. Disease-specific survival was defined as the proportion of patients not deceased from uveal melanoma, according to data in the Cause of Death Register and/or our clinical records as described above. Median follow-up was defined as the median time (years) from plaque brachytherapy to last occasion surviving patients were seen or in contact. The relative survival rate was defined as the observed cumulative proportion of uveal melanoma patients surviving divided with the expected cumulative proportion surviving in the general population at the same point in time^23.^ 95% CI of the relative survival rate was calculated by dividing the standard error of the observed cumulative survival rate by the expected survival rate^19^:

$$95 \%\,CI= Relative\,survival\, rate \pm 1.96\times \frac{standard\,error\,of\,the\,observed\,survival\,rate}{Expected\,survival\,rate}$$

All statistical analyses were performed using SPSS statistics version 26 (IBM, Armonk, NY, USA).

## Supplementary information

Supplementary file1 (PDF 1334 kb)
