# Supplementary material for: Forty-year prognosis after plaque brachytherapy of uveal melanoma
Source: Sci Rep. 2020 Jul 9;10:11297. doi: 10.1038/s41598-020-68232-7 (PMC7347921; doi:10.1038/s41598-020-68232-7)
Supplement: Supplementary file 1 — Supplementary file1 (PDF 1334 kb) [file 41598_2020_68232_MOESM1_ESM.pdf]

Supplementary material to:

Forty-year prognosis after plaque brachytherapy of uveal melanoma

Gustav Stålhammar[1,2]

[1]St. Erik Eye Hospital, Stockholm, 11282, Sweden
[2]Department of Clinical Neuroscience, Karolinska Institutet, Stockholm, 17177, Sweden

Corresponding author:
Gustav Stålhammar
Associate professor
M.D. Ph.D. FEBO
St. Erik Eye Hospital
Polhemsgatan 50
112 82 Stockholm, Sweden
Phone: 0046 8 672 30 00
Email: gustav.stalhammar@ki.se

Supplementary table 1. Remaining life expectancy for **men** in the general population, based on calendar year and patient age at plaque brachytherapy.

		**Year of diagnosis**																			
		**1980**	**1981**	**1982**	**1983**	**1984**	**1985**	**1986**	**1987**	**1988**	**1989**	**1990**	**1991**	**1992**	**1993**	**1994**	**1995**	**1996**	**1997**	**1998**	**1999**
**Age at diagnosis**	**0 years**	72,8	73,1	73,4	73,6	73,8	73,8	74,0	74,2	74,2	74,8	74,8	74,9	75,4	75,5	76,1	76,2	76,5	76,7	76,9	77,1
	**1 year**	72,4	72,6	73,0	73,1	73,4	73,3	73,5	73,7	73,6	74,3	74,3	74,4	74,8	74,9	75,5	75,5	75,8	76,0	76,2	76,4
	**2 years**	71,4	71,6	72,0	72,2	72,4	72,4	72,5	72,7	72,7	73,3	73,3	73,5	73,8	73,9	74,5	74,6	74,9	75,0	75,2	75,4
	**3 years**	70,4	70,6	71,0	71,2	71,4	71,4	71,5	71,7	71,7	72,3	72,4	72,5	72,9	73,0	73,5	73,6	73,9	74,1	74,2	74,4
	**4 years**	69,4	69,7	70,0	70,2	70,4	70,4	70,5	70,7	70,7	71,3	71,4	71,5	71,9	72,0	72,5	72,6	72,9	73,1	73,3	73,4
	**5 years**	68,5	68,7	69,1	69,2	69,4	69,4	69,5	69,8	69,7	70,4	70,4	70,5	70,9	71,0	71,5	71,6	71,9	72,1	72,3	72,4
	**6 years**	67,5	67,7	68,1	68,2	68,5	68,4	68,6	68,8	68,7	69,4	69,4	69,5	69,9	70,0	70,5	70,6	70,9	71,1	71,3	71,4
	**7 years**	66,5	66,7	67,1	67,3	67,5	67,5	67,6	67,8	67,8	68,4	68,4	68,5	68,9	69,0	69,6	69,6	69,9	70,1	70,3	70,4
	**8 years**	65,5	65,7	66,1	66,3	66,5	66,5	66,6	66,8	66,8	67,4	67,4	67,5	67,9	68,0	68,6	68,6	68,9	69,1	69,3	69,5
	**9 years**	64,5	64,8	65,1	65,3	65,5	65,5	65,6	65,8	65,8	66,4	66,5	66,6	66,9	67,0	67,6	67,6	67,9	68,1	68,3	68,5
	**10 years**	63,5	63,8	64,1	64,3	64,5	64,5	64,6	64,8	64,8	65,4	65,5	65,6	65,9	66,1	66,6	66,6	67,0	67,1	67,3	67,5
	**11 years**	62,6	62,8	63,1	63,3	63,5	63,5	63,6	63,8	63,8	64,4	64,5	64,6	65,0	65,1	65,6	65,6	66,0	66,2	66,3	66,5
	**12 years**	61,6	61,8	62,1	62,3	62,5	62,5	62,7	62,8	62,8	63,4	63,5	63,6	64,0	64,1	64,6	64,7	65,0	65,2	65,3	65,5
	**13 years**	60,6	60,8	61,2	61,3	61,5	61,5	61,7	61,8	61,8	62,4	62,5	62,6	63,0	63,1	63,6	63,7	64,0	64,2	64,3	64,5
	**14 years**	59,6	59,8	60,2	60,4	60,6	60,5	60,7	60,9	60,8	61,5	61,5	61,6	62,0	62,1	62,6	62,7	63,0	63,2	63,4	63,5
	**15 years**	58,6	58,8	59,2	59,4	59,6	59,5	59,7	59,9	59,9	60,5	60,5	60,6	61,0	61,1	61,6	61,7	62,0	62,2	62,4	62,5
	**16 years**	57,6	57,9	58,2	58,4	58,6	58,6	58,7	58,9	58,9	59,5	59,5	59,6	60,0	60,1	60,6	60,7	61,0	61,2	61,4	61,5
	**17 years**	56,7	56,9	57,2	57,4	57,6	57,6	57,8	57,9	57,9	58,5	58,6	58,7	59,0	59,1	59,7	59,7	60,0	60,2	60,4	60,6

	**18 years**	55,7	55,9	56,3	56,4	56,6	56,6	56,8	57,0	57,0	57,5	57,6	57,7	58,1	58,2	58,7	58,7	59,1	59,2	59,5	59,6
	**19 years**	54,7	55,0	55,3	55,5	55,7	55,7	55,8	56,0	56,0	56,6	56,6	56,7	57,1	57,2	57,7	57,8	58,1	58,2	58,5	58,6
	**20 years**	53,8	54,0	54,3	54,6	54,7	54,7	54,9	55,1	55,0	55,7	55,7	55,8	56,1	56,2	56,7	56,8	57,1	57,3	57,5	57,6
	**21 years**	52,8	53,1	53,4	53,6	53,8	53,8	53,9	54,1	54,1	54,7	54,7	54,8	55,2	55,3	55,8	55,9	56,1	56,3	56,6	56,7
	**22 years**	51,9	52,1	52,4	52,6	52,8	52,8	53,0	53,2	53,1	53,8	53,8	53,9	54,2	54,3	54,8	54,9	55,2	55,3	55,6	55,7
	**23 years**	50,9	51,2	51,5	51,7	51,9	51,9	52,0	52,2	52,2	52,8	52,8	52,9	53,2	53,4	53,8	53,9	54,2	54,4	54,6	54,8
	**24 years**	50,0	50,2	50,5	50,8	51,0	50,9	51,1	51,3	51,2	51,9	51,9	51,9	52,3	52,4	52,9	53,0	53,2	53,4	53,7	53,8
	**25 years**	49,0	49,3	49,6	49,8	50,0	49,9	50,1	50,3	50,3	50,9	50,9	51,0	51,3	51,4	51,9	52,0	52,3	52,5	52,7	52,8
	**26 years**	48,1	48,3	48,6	48,9	49,1	49,0	49,2	49,4	49,3	49,9	50,0	50,0	50,4	50,5	51,0	51,0	51,3	51,5	51,7	51,9
	**27 years**	47,2	47,4	47,7	47,9	48,1	48,0	48,2	48,4	48,4	49,0	49,0	49,1	49,4	49,5	50,0	50,1	50,3	50,5	50,8	50,9
	**28 years**	46,2	46,4	46,7	47,0	47,2	47,1	47,3	47,5	47,4	48,1	48,1	48,1	48,4	48,6	49,0	49,1	49,4	49,6	49,8	49,9
	**29 years**	45,3	45,5	45,8	46,0	46,2	46,1	46,3	46,5	46,5	47,1	47,1	47,2	47,5	47,6	48,1	48,1	48,4	48,6	48,8	49,0
	**30 years**	44,3	44,5	44,8	45,1	45,3	45,2	45,4	45,6	45,5	46,2	46,2	46,2	46,5	46,6	47,1	47,2	47,4	47,6	47,9	48,0
	**31 years**	43,4	43,6	43,9	44,1	44,3	44,2	44,4	44,6	44,6	45,2	45,2	45,3	45,6	45,7	46,2	46,2	46,5	46,7	46,9	47,0
	**32 years**	42,4	42,6	42,9	43,2	43,4	43,3	43,5	43,7	43,6	44,3	44,3	44,3	44,6	44,7	45,2	45,3	45,5	45,7	45,9	46,1
	**33 years**	41,5	41,7	42,0	42,2	42,4	42,3	42,5	42,7	42,7	43,3	43,3	43,4	43,7	43,8	44,3	44,3	44,6	44,7	44,9	45,1
	**34 years**	40,6	40,7	41,0	41,3	41,5	41,4	41,6	41,8	41,8	42,4	42,3	42,4	42,7	42,8	43,3	43,3	43,6	43,8	44,0	44,1
	**35 years**	39,6	39,8	40,1	40,3	40,5	40,4	40,6	40,8	40,8	41,4	41,4	41,5	41,8	41,8	42,3	42,4	42,6	42,8	43,0	43,2
	**36 years**	38,7	38,8	39,1	39,4	39,6	39,5	39,7	39,9	39,9	40,5	40,4	40,5	40,8	40,9	41,4	41,4	41,7	41,9	42,1	42,2
	**37 years**	37,8	37,9	38,2	38,4	38,6	38,5	38,7	38,9	38,9	39,5	39,5	39,6	39,9	40,0	40,4	40,5	40,7	40,9	41,1	41,3
	**38 years**	36,8	37,0	37,3	37,5	37,7	37,6	37,8	38,0	38,0	38,6	38,5	38,6	38,9	39,0	39,5	39,5	39,8	40,0	40,2	40,3

	**39 years**	35,9	36,0	36,3	36,5	36,8	36,6	36,9	37,1	37,1	37,7	37,6	37,7	38,0	38,1	38,6	38,6	38,8	39,0	39,2	39,3
	**40 years**	34,9	35,1	35,4	35,6	35,8	35,7	35,9	36,1	36,1	36,7	36,7	36,8	37,0	37,1	37,6	37,6	37,9	38,1	38,3	38,4
	**41 years**	34,0	34,2	34,5	34,7	34,9	34,8	35,0	35,2	35,2	35,8	35,7	35,8	36,1	36,2	36,7	36,7	36,9	37,1	37,3	37,5
	**42 years**	33,1	33,3	33,5	33,7	34,0	33,9	34,1	34,2	34,3	34,9	34,8	34,9	35,1	35,3	35,8	35,8	36,0	36,2	36,4	36,5
	**43 years**	32,2	32,3	32,6	32,8	33,0	32,9	33,1	33,3	33,3	33,9	33,9	34,0	34,2	34,3	34,8	34,8	35,1	35,2	35,4	35,6
	**44 years**	31,3	31,4	31,7	31,9	32,1	32,0	32,2	32,4	32,4	33,0	32,9	33,0	33,3	33,4	33,9	33,9	34,1	34,3	34,5	34,7
	**45 years**	30,4	30,5	30,8	31,0	31,2	31,1	31,3	31,5	31,5	32,1	32,0	32,1	32,4	32,5	33,0	33,0	33,2	33,4	33,5	33,7
	**46 years**	29,5	29,6	29,9	30,1	30,3	30,2	30,4	30,6	30,6	31,2	31,1	31,2	31,5	31,6	32,1	32,1	32,3	32,4	32,6	32,8
	**47 years**	28,6	28,7	29,0	29,2	29,4	29,3	29,5	29,6	29,7	30,3	30,2	30,3	30,5	30,6	31,2	31,1	31,3	31,5	31,7	31,9
	**48 years**	27,8	27,9	28,1	28,3	28,5	28,4	28,6	28,8	28,8	29,4	29,3	29,4	29,6	29,7	30,2	30,2	30,4	30,6	30,8	30,9
	**49 years**	26,9	27,0	27,2	27,4	27,6	27,5	27,7	27,9	27,9	28,5	28,4	28,5	28,7	28,8	29,3	29,3	29,5	29,7	29,9	30,0
	**50 years**	26,0	26,1	26,4	26,5	26,7	26,6	26,8	27,0	27,0	27,6	27,5	27,6	27,8	27,9	28,4	28,4	28,6	28,8	28,9	29,1
	**51 years**	25,2	25,2	25,5	25,6	25,9	25,7	26,0	26,1	26,1	26,7	26,6	26,7	26,9	27,0	27,5	27,5	27,7	27,9	28,0	28,2
	**52 years**	24,3	24,4	24,7	24,8	25,0	24,9	25,1	25,3	25,2	25,8	25,7	25,8	26,0	26,1	26,7	26,6	26,8	27,0	27,1	27,3
	**53 years**	23,5	23,6	23,8	24,0	24,2	24,0	24,2	24,4	24,4	24,9	24,9	25,0	25,2	25,3	25,8	25,8	26,0	26,1	26,2	26,4
	**54 years**	22,6	22,7	23,0	23,1	23,3	23,2	23,4	23,6	23,5	24,1	24,0	24,1	24,3	24,4	24,9	24,9	25,1	25,2	25,4	25,5
	**55 years**	21,8	21,9	22,1	22,3	22,5	22,3	22,5	22,7	22,7	23,2	23,1	23,2	23,4	23,5	24,0	24,0	24,2	24,4	24,5	24,7
	**56 years**	21,0	21,1	21,3	21,5	21,6	21,5	21,7	21,9	21,9	22,4	22,3	22,4	22,6	22,7	23,2	23,2	23,4	23,5	23,6	23,8
	**57 years**	20,2	20,3	20,5	20,6	20,9	20,7	20,9	21,1	21,1	21,6	21,5	21,6	21,8	21,9	22,3	22,3	22,5	22,6	22,8	23,0
	**58 years**	19,4	19,5	19,7	19,8	20,0	19,9	20,1	20,3	20,2	20,8	20,7	20,8	21,0	21,0	21,5	21,5	21,7	21,8	21,9	22,1
	**59 years**	18,6	18,7	19,0	19,1	19,3	19,1	19,3	19,5	19,4	20,0	19,9	20,0	20,1	20,2	20,7	20,6	20,8	21,0	21,1	21,3

	**60 years**	17,9	18,0	18,2	18,3	18,5	18,3	18,5	18,7	18,6	19,2	19,1	19,2	19,3	19,4	19,9	19,8	20,0	20,1	20,3	20,4
	**61 years**	17,2	17,2	17,4	17,5	17,7	17,6	17,8	18,0	17,9	18,4	18,3	18,4	18,5	18,6	19,1	19,0	19,2	19,3	19,4	19,6
	**62 years**	16,4	16,5	16,7	16,8	17,0	16,8	17,0	17,2	17,1	17,6	17,5	17,6	17,8	17,8	18,3	18,3	18,4	18,6	18,6	18,8
	**63 years**	15,7	15,7	16,0	16,1	16,2	16,1	16,2	16,4	16,4	16,9	16,8	16,9	17,0	17,1	17,5	17,5	17,6	17,8	17,9	18,0
	**64 years**	15,0	15,0	15,3	15,4	15,5	15,4	15,5	15,7	15,7	16,1	16,0	16,1	16,3	16,3	16,8	16,7	16,8	17,0	17,1	17,2
	**65 years**	14,3	14,3	14,6	14,7	14,8	14,7	14,8	15,0	15,0	15,4	15,3	15,4	15,6	15,6	16,0	16,0	16,1	16,2	16,3	16,5
	**66 years**	13,6	13,7	13,9	14,0	14,1	14,0	14,1	14,3	14,2	14,7	14,6	14,7	14,9	14,8	15,3	15,2	15,4	15,5	15,6	15,7
	**67 years**	13,0	13,0	13,2	13,3	13,5	13,3	13,5	13,6	13,6	14,0	13,9	14,0	14,2	14,1	14,6	14,5	14,7	14,8	14,9	14,9
	**68 years**	12,3	12,4	12,6	12,7	12,8	12,7	12,8	13,0	12,9	13,3	13,2	13,3	13,5	13,5	13,9	13,8	14,0	14,1	14,2	14,2
	**69 years**	11,7	11,7	12,0	12,0	12,2	12,0	12,2	12,3	12,3	12,7	12,5	12,7	12,8	12,8	13,2	13,1	13,3	13,4	13,5	13,5
	**70 years**	11,1	11,2	11,4	11,4	11,5	11,4	11,5	11,7	11,6	12,0	11,9	12,0	12,1	12,1	12,5	12,5	12,6	12,7	12,8	12,8
	**71 years**	10,5	10,6	10,8	10,8	10,9	10,8	10,9	11,0	11,0	11,4	11,3	11,4	11,5	11,5	11,9	11,8	11,9	12,0	12,1	12,2
	**72 years**	9,9	10,0	10,2	10,2	10,3	10,2	10,3	10,5	10,4	10,8	10,6	10,8	10,9	10,8	11,3	11,2	11,3	11,4	11,5	11,5
	**73 years**	9,4	9,4	9,6	9,6	9,8	9,7	9,8	9,9	9,8	10,2	10,1	10,2	10,3	10,2	10,7	10,5	10,7	10,8	10,9	10,9
	**74 years**	8,9	8,9	9,1	9,1	9,2	9,1	9,2	9,3	9,3	9,6	9,5	9,6	9,7	9,7	10,1	9,9	10,1	10,2	10,2	10,3
	**75 years**	8,4	8,4	8,6	8,6	8,7	8,6	8,7	8,8	8,7	9,1	8,9	9,0	9,2	9,1	9,5	9,4	9,5	9,6	9,6	9,7
	**76 years**	7,9	7,9	8,1	8,1	8,2	8,1	8,2	8,3	8,2	8,6	8,4	8,5	8,6	8,5	8,9	8,8	8,9	9,0	9,1	9,1
	**77 years**	7,4	7,4	7,6	7,6	7,7	7,6	7,7	7,8	7,7	8,1	7,9	8,0	8,1	8,0	8,4	8,3	8,4	8,5	8,5	8,5
	**78 years**	7,0	7,0	7,1	7,2	7,3	7,1	7,2	7,3	7,2	7,6	7,4	7,5	7,6	7,5	7,9	7,8	7,9	8,0	8,0	8,0
	**79 years**	6,5	6,6	6,7	6,7	6,9	6,7	6,8	6,9	6,8	7,1	7,0	7,1	7,1	7,1	7,5	7,3	7,3	7,5	7,5	7,5
	**80 years**	6,1	6,2	6,3	6,3	6,5	6,3	6,4	6,5	6,3	6,7	6,6	6,6	6,7	6,6	7,0	6,9	6,9	7,0	7,0	7,0

	**81 years**	5,8	5,8	6,0	5,9	6,1	5,9	6,0	6,1	6,0	6,3	6,1	6,2	6,3	6,2	6,6	6,4	6,4	6,5	6,5	6,5
	**82 years**	5,4	5,4	5,6	5,5	5,7	5,5	5,6	5,7	5,6	5,9	5,8	5,8	5,8	5,8	6,2	6,0	6,0	6,1	6,1	6,1
	**83 years**	5,1	5,1	5,2	5,2	5,3	5,2	5,2	5,3	5,2	5,5	5,4	5,4	5,5	5,4	5,8	5,6	5,6	5,7	5,6	5,7
	**84 years**	4,7	4,7	4,9	4,8	5,0	4,9	4,9	4,9	4,9	5,1	5,0	5,1	5,1	5,0	5,4	5,2	5,2	5,3	5,3	5,3
	**85 years**	4,4	4,5	4,7	4,5	4,7	4,5	4,6	4,6	4,6	4,8	4,7	4,7	4,8	4,7	5,0	4,9	4,9	4,9	4,9	4,9
	**86 years**	4,1	4,2	4,4	4,3	4,4	4,2	4,2	4,3	4,3	4,5	4,4	4,4	4,5	4,3	4,7	4,5	4,5	4,6	4,6	4,5
	**87 years**	3,9	3,9	4,1	3,9	4,0	3,9	3,9	4,0	4,0	4,2	4,1	4,1	4,1	4,0	4,4	4,3	4,2	4,3	4,3	4,2
	**88 years**	3,6	3,7	3,8	3,7	3,7	3,7	3,7	3,8	3,7	4,0	3,8	3,8	3,9	3,8	4,1	4,0	4,0	4,0	4,0	3,9
	**89 years**	3,3	3,4	3,6	3,4	3,4	3,5	3,5	3,5	3,5	3,7	3,6	3,6	3,6	3,5	3,8	3,7	3,7	3,7	3,7	3,6
	**90 years**	3,0	3,2	3,4	3,3	3,2	3,2	3,2	3,3	3,3	3,4	3,4	3,3	3,3	3,3	3,6	3,5	3,5	3,4	3,4	3,4
	**91 years**	2,9	2,9	3,1	3,0	3,0	3,0	3,0	3,1	3,1	3,2	3,1	3,1	3,1	3,1	3,3	3,2	3,2	3,2	3,2	3,2
	**92 years**	2,6	2,7	2,9	2,8	2,7	2,8	2,7	2,9	2,9	3,0	2,9	2,9	2,9	2,9	3,1	3,0	3,0	3,0	3,0	2,9
	**93 years**	2,4	2,5	2,7	2,6	2,5	2,6	2,5	2,7	2,7	2,8	2,7	2,7	2,8	2,7	2,9	2,8	2,8	2,8	2,8	2,8
	**94 years**	2,2	2,3	2,5	2,4	2,3	2,4	2,3	2,5	2,5	2,6	2,6	2,6	2,6	2,5	2,7	2,7	2,6	2,6	2,6	2,6
	**95 years**	2,0	2,1	2,3	2,2	2,1	2,2	2,1	2,4	2,4	2,5	2,4	2,4	2,4	2,4	2,5	2,5	2,5	2,5	2,5	2,4
	**96 years**	1,8	1,9	2,1	2,0	1,9	2,0	1,9	2,2	2,2	2,3	2,3	2,3	2,3	2,2	2,4	2,3	2,3	2,3	2,3	2,3
	**97 years**	1,7	1,7	1,9	1,8	1,7	1,8	1,7	2,1	2,1	2,2	2,1	2,1	2,1	2,1	2,2	2,2	2,2	2,2	2,2	2,1
	**98 years**	1,5	1,5	1,7	1,6	1,6	1,6	1,6	2,0	2,0	2,0	2,0	2,0	2,0	2,0	2,1	2,1	2,0	2,0	2,0	2,0
	**99 years**	1,3	1,3	1,5	1,4	1,4	1,4	1,4	1,8	1,8	1,9	1,9	1,9	1,9	1,8	2,0	1,9	1,9	1,9	1,9	1,9
	**100 years**	1,2	1,2	1,3	1,3	1,3	1,3	1,2	1,7	1,7	1,8	1,8	1,8	1,8	1,7	1,8	1,8	1,8	1,8	1,8	1,8

Supplementary table 2. Remaining life expectancy for **women** in the general population, based on calendar year and patient age at plaque brachytherapy.

		**Year of diagnosis**																			
		**1980**	**1981**	**1982**	**1983**	**1984**	**1985**	**1986**	**1987**	**1988**	**1989**	**1990**	**1991**	**1992**	**1993**	**1994**	**1995**	**1996**	**1997**	**1998**	**1999**
**Age at diag nosis**	**0 years**	78,8	79,1	79,4	79,6	79,9	79,7	80,0	80,2	80,0	80,6	80,4	80,5	80,8	80,8	81,4	81,5	81,5	81,8	81,9	81,9
	**1 year**	78,3	78,6	78,9	79,2	79,3	79,2	79,4	79,6	79,4	80,0	79,8	80,0	80,2	80,1	80,7	80,7	80,8	81,1	81,2	81,1
	**2 years**	77,3	77,6	77,9	78,2	78,4	78,2	78,5	78,6	78,4	79,0	78,9	79,0	79,2	79,2	79,7	79,8	79,9	80,1	80,2	80,2
	**3 years**	76,3	76,6	77,0	77,2	77,4	77,3	77,5	77,7	77,4	78,0	77,9	78,1	78,2	78,2	78,7	78,8	78,9	79,1	79,2	79,2
	**4 years**	75,3	75,7	76,0	76,2	76,4	76,3	76,5	76,7	76,4	77,0	76,9	77,1	77,2	77,2	77,7	77,8	77,9	78,2	78,2	78,2
	**5 years**	74,4	74,7	75,0	75,2	75,4	75,3	75,5	75,7	75,4	76,1	75,9	76,1	76,2	76,2	76,8	76,8	76,9	77,2	77,2	77,2
	**6 years**	73,4	73,7	74,0	74,3	74,4	74,3	74,5	74,7	74,5	75,1	74,9	75,1	75,2	75,2	75,8	75,8	75,9	76,2	76,2	76,2
	**7 years**	72,4	72,7	73,0	73,3	73,4	73,3	73,5	73,7	73,5	74,1	73,9	74,1	74,3	74,2	74,8	74,8	74,9	75,2	75,2	75,2
	**8 years**	71,4	71,7	72,0	72,3	72,4	72,3	72,6	72,7	72,5	73,1	72,9	73,1	73,3	73,2	73,8	73,8	73,9	74,2	74,3	74,2
	**9 years**	70,4	70,7	71,0	71,3	71,4	71,3	71,6	71,7	71,5	72,1	71,9	72,1	72,3	72,2	72,8	72,8	72,9	73,2	73,3	73,2
	**10 years**	69,4	69,7	70,0	70,3	70,5	70,3	70,6	70,7	70,5	71,1	71,0	71,1	71,3	71,2	71,8	71,8	71,9	72,2	72,3	72,2
	**11 years**	68,4	68,7	69,1	69,3	69,5	69,3	69,6	69,8	69,5	70,1	70,0	70,1	70,3	70,3	70,8	70,8	70,9	71,2	71,3	71,2
	**12 years**	67,4	67,7	68,1	68,3	68,5	68,3	68,6	68,8	68,5	69,1	69,0	69,1	69,3	69,3	69,8	69,8	69,9	70,2	70,3	70,2
	**13 years**	66,5	66,7	67,1	67,3	67,5	67,3	67,6	67,8	67,5	68,1	68,0	68,2	68,3	68,3	68,8	68,8	68,9	69,2	69,3	69,2
	**14 years**	65,5	65,7	66,1	66,3	66,5	66,4	66,6	66,8	66,5	67,1	67,0	67,2	67,3	67,3	67,8	67,9	68,0	68,2	68,3	68,2
	**15 years**	64,5	64,8	65,1	65,3	65,5	65,4	65,6	65,8	65,5	66,1	66,0	66,2	66,3	66,3	66,8	66,9	67,0	67,2	67,3	67,3
	**16 years**	63,5	63,8	64,1	64,3	64,5	64,4	64,6	64,8	64,6	65,2	65,0	65,2	65,3	65,3	65,8	65,9	66,0	66,2	66,3	66,3
	**17 years**	62,5	62,8	63,1	63,4	63,5	63,4	63,6	63,8	63,6	64,2	64,0	64,2	64,4	64,3	64,8	64,9	65,0	65,3	65,4	65,3

	**18 years**	61,5	61,8	62,2	62,4	62,5	62,4	62,7	62,8	62,6	63,2	63,1	63,2	63,4	63,3	63,9	63,9	64,0	64,3	64,4	64,3
	**19 years**	60,6	60,8	61,2	61,4	61,6	61,4	61,7	61,8	61,6	62,2	62,1	62,2	62,4	62,4	62,9	62,9	63,0	63,3	63,4	63,3
	**20 years**	59,6	59,9	60,2	60,4	60,6	60,5	60,7	60,9	60,6	61,2	61,1	61,3	61,4	61,4	61,9	61,9	62,0	62,3	62,4	62,3
	**21 years**	58,6	58,9	59,2	59,4	59,6	59,5	59,7	59,9	59,6	60,3	60,1	60,3	60,4	60,4	60,9	61,0	61,0	61,3	61,4	61,3
	**22 years**	57,6	57,9	58,2	58,5	58,6	58,5	58,7	58,9	58,7	59,3	59,1	59,3	59,4	59,4	59,9	60,0	60,1	60,3	60,4	60,3
	**23 years**	56,7	56,9	57,2	57,5	57,6	57,5	57,7	57,9	57,7	58,3	58,1	58,3	58,5	58,4	58,9	59,0	59,1	59,4	59,5	59,4
	**24 years**	55,7	55,9	56,3	56,5	56,7	56,5	56,8	56,9	56,7	57,3	57,2	57,3	57,5	57,5	57,9	58,0	58,1	58,4	58,5	58,4
	**25 years**	54,7	55,0	55,3	55,5	55,7	55,6	55,8	56,0	55,7	56,3	56,2	56,3	56,5	56,5	57,0	57,0	57,1	57,4	57,5	57,4
	**26 years**	53,7	54,0	54,3	54,5	54,7	54,6	54,8	55,0	54,8	55,4	55,2	55,4	55,5	55,5	56,0	56,0	56,1	56,4	56,5	56,4
	**27 years**	52,7	53,0	53,3	53,6	53,7	53,6	53,8	54,0	53,8	54,4	54,2	54,4	54,5	54,5	55,0	55,1	55,1	55,4	55,5	55,4
	**28 years**	51,8	52,0	52,3	52,6	52,7	52,6	52,8	53,0	52,8	53,4	53,3	53,4	53,5	53,5	54,0	54,1	54,2	54,4	54,5	54,4
	**29 years**	50,8	51,0	51,4	51,6	51,8	51,7	51,9	52,1	51,8	52,4	52,3	52,4	52,6	52,6	53,0	53,1	53,2	53,5	53,5	53,5
	**30 years**	49,8	50,1	50,4	50,6	50,8	50,7	50,9	51,1	50,9	51,4	51,3	51,4	51,6	51,6	52,1	52,1	52,2	52,5	52,6	52,5
	**31 years**	48,8	49,1	49,4	49,7	49,8	49,7	49,9	50,1	49,9	50,5	50,3	50,5	50,6	50,6	51,1	51,1	51,2	51,5	51,6	51,5
	**32 years**	47,9	48,1	48,4	48,7	48,8	48,7	49,0	49,1	48,9	49,5	49,4	49,5	49,6	49,6	50,1	50,2	50,2	50,5	50,6	50,5
	**33 years**	46,9	47,2	47,5	47,7	47,9	47,8	48,0	48,2	47,9	48,5	48,4	48,5	48,6	48,6	49,1	49,2	49,2	49,5	49,6	49,5
	**34 years**	45,9	46,2	46,5	46,8	46,9	46,8	47,0	47,2	47,0	47,5	47,4	47,5	47,7	47,7	48,2	48,2	48,3	48,5	48,6	48,5
	**35 years**	45,0	45,2	45,5	45,8	45,9	45,8	46,0	46,2	46,0	46,6	46,4	46,6	46,7	46,7	47,2	47,2	47,3	47,6	47,6	47,6
	**36 years**	44,0	44,3	44,6	44,8	45,0	44,9	45,1	45,3	45,0	45,6	45,5	45,6	45,7	45,7	46,2	46,2	46,3	46,6	46,7	46,6
	**37 years**	43,1	43,3	43,6	43,9	44,0	43,9	44,1	44,3	44,1	44,6	44,5	44,6	44,8	44,8	45,2	45,3	45,4	45,6	45,7	45,6
	**38 years**	42,1	42,3	42,6	42,9	43,0	43,0	43,1	43,3	43,1	43,7	43,5	43,7	43,8	43,8	44,3	44,3	44,4	44,7	44,7	44,6

	**39 years**	41,1	41,4	41,7	41,9	42,1	42,0	42,2	42,4	42,2	42,7	42,6	42,7	42,8	42,8	43,3	43,3	43,4	43,7	43,8	43,7
	**40 years**	40,2	40,4	40,7	41,0	41,1	41,0	41,2	41,4	41,2	41,8	41,6	41,8	41,8	41,9	42,4	42,4	42,4	42,7	42,8	42,7
	**41 years**	39,2	39,4	39,8	40,0	40,2	40,1	40,3	40,4	40,2	40,8	40,6	40,8	40,9	40,9	41,4	41,4	41,5	41,7	41,8	41,7
	**42 years**	38,3	38,5	38,8	39,1	39,2	39,1	39,3	39,5	39,3	39,9	39,7	39,8	39,9	39,9	40,4	40,4	40,5	40,8	40,9	40,8
	**43 years**	37,4	37,5	37,9	38,1	38,3	38,2	38,4	38,5	38,3	38,9	38,7	38,9	39,0	39,0	39,5	39,5	39,5	39,8	39,9	39,8
	**44 years**	36,4	36,6	36,9	37,2	37,3	37,2	37,4	37,6	37,4	38,0	37,8	37,9	38,0	38,0	38,5	38,5	38,6	38,9	39,0	38,9
	**45 years**	35,5	35,6	36,0	36,2	36,4	36,3	36,5	36,6	36,5	37,0	36,9	37,0	37,1	37,1	37,6	37,6	37,6	37,9	38,0	37,9
	**46 years**	34,6	34,7	35,0	35,3	35,4	35,3	35,5	35,7	35,5	36,1	35,9	36,1	36,1	36,1	36,6	36,6	36,7	37,0	37,1	37,0
	**47 years**	33,7	33,8	34,1	34,3	34,5	34,4	34,6	34,8	34,6	35,1	35,0	35,1	35,2	35,2	35,7	35,7	35,8	36,0	36,1	36,0
	**48 years**	32,7	32,9	33,2	33,4	33,6	33,5	33,7	33,9	33,7	34,2	34,0	34,2	34,3	34,3	34,8	34,8	34,8	35,1	35,2	35,1
	**49 years**	31,8	31,9	32,3	32,5	32,6	32,5	32,8	32,9	32,7	33,3	33,1	33,3	33,3	33,3	33,9	33,8	33,9	34,1	34,2	34,2
	**50 years**	30,9	31,0	31,3	31,6	31,7	31,6	31,8	32,0	31,9	32,4	32,2	32,3	32,4	32,4	32,9	32,9	33,0	33,2	33,3	33,2
	**51 years**	30,0	30,1	30,4	30,7	30,8	30,7	30,9	31,1	30,9	31,5	31,3	31,4	31,5	31,5	32,0	32,0	32,0	32,3	32,4	32,3
	**52 years**	29,1	29,2	29,5	29,8	29,9	29,8	30,0	30,2	30,0	30,5	30,4	30,5	30,6	30,6	31,1	31,1	31,1	31,4	31,5	31,4
	**53 years**	28,2	28,3	28,6	28,9	29,0	28,9	29,1	29,3	29,1	29,6	29,5	29,6	29,7	29,6	30,2	30,2	30,2	30,5	30,5	30,5
	**54 years**	27,3	27,4	27,7	28,0	28,1	28,0	28,2	28,4	28,2	28,7	28,6	28,7	28,8	28,7	29,3	29,3	29,3	29,5	29,6	29,5
	**55 years**	26,4	26,5	26,8	27,1	27,2	27,1	27,3	27,5	27,3	27,8	27,6	27,8	27,9	27,8	28,4	28,4	28,4	28,6	28,7	28,6
	**56 years**	25,5	25,6	26,0	26,2	26,3	26,2	26,4	26,6	26,4	26,9	26,8	26,9	27,0	26,9	27,5	27,5	27,5	27,7	27,8	27,7
	**57 years**	24,6	24,7	25,1	25,3	25,4	25,3	25,5	25,7	25,5	26,0	25,9	26,0	26,1	26,0	26,6	26,6	26,6	26,8	26,9	26,9
	**58 years**	23,8	23,9	24,2	24,4	24,6	24,4	24,6	24,8	24,6	25,2	25,0	25,1	25,2	25,2	25,7	25,7	25,7	25,9	26,0	26,0
	**59 years**	22,9	23,0	23,4	23,6	23,7	23,6	23,8	24,0	23,8	24,3	24,1	24,3	24,3	24,3	24,9	24,8	24,9	25,1	25,2	25,1

	**60 years**	22,1	22,2	22,5	22,7	22,8	22,7	22,9	23,1	22,9	23,4	23,3	23,4	23,5	23,4	24,0	23,9	24,0	24,2	24,3	24,2
	**61 years**	21,2	21,3	21,7	21,8	22,0	21,9	22,0	22,2	22,1	22,6	22,4	22,6	22,6	22,6	23,1	23,0	23,1	23,3	23,4	23,3
	**62 years**	20,4	20,5	20,8	21,0	21,1	21,0	21,2	21,4	21,2	21,7	21,6	21,7	21,8	21,7	22,3	22,2	22,3	22,5	22,5	22,4
	**63 years**	19,6	19,6	20,0	20,1	20,3	20,2	20,3	20,5	20,4	20,8	20,7	20,9	20,9	20,9	21,4	21,4	21,4	21,6	21,7	21,6
	**64 years**	18,7	18,8	19,1	19,3	19,5	19,3	19,5	19,7	19,5	20,0	19,9	20,0	20,1	20,0	20,6	20,5	20,6	20,7	20,9	20,8
	**65 years**	17,9	18,0	18,3	18,5	18,7	18,5	18,7	18,9	18,7	19,2	19,0	19,2	19,3	19,2	19,8	19,7	19,7	19,9	20,0	19,9
	**66 years**	17,1	17,2	17,5	17,7	17,8	17,7	17,9	18,1	17,9	18,4	18,2	18,4	18,5	18,4	18,9	18,9	18,9	19,1	19,2	19,1
	**67 years**	16,4	16,4	16,7	16,9	17,0	16,9	17,1	17,3	17,1	17,6	17,4	17,6	17,7	17,6	18,1	18,1	18,1	18,3	18,4	18,3
	**68 years**	15,6	15,6	16,0	16,1	16,2	16,1	16,3	16,5	16,3	16,8	16,6	16,8	16,9	16,8	17,3	17,3	17,3	17,5	17,6	17,5
	**69 years**	14,8	14,8	15,2	15,3	15,5	15,3	15,5	15,7	15,5	16,0	15,9	16,0	16,1	16,0	16,5	16,5	16,5	16,7	16,8	16,7
	**70 years**	14,0	14,1	14,4	14,6	14,7	14,6	14,8	14,9	14,8	15,2	15,1	15,2	15,3	15,2	15,7	15,7	15,7	15,9	16,0	15,9
	**71 years**	13,3	13,4	13,7	13,8	13,9	13,8	14,0	14,2	14,0	14,4	14,3	14,5	14,6	14,5	14,9	14,9	14,9	15,1	15,2	15,1
	**72 years**	12,6	12,7	13,0	13,1	13,2	13,1	13,3	13,5	13,3	13,7	13,6	13,8	13,8	13,7	14,2	14,2	14,2	14,3	14,4	14,3
	**73 years**	11,9	12,0	12,3	12,4	12,5	12,4	12,6	12,8	12,6	12,9	12,8	13,0	13,1	13,0	13,5	13,4	13,4	13,6	13,7	13,6
	**74 years**	11,2	11,3	11,6	11,7	11,8	11,7	11,8	12,0	11,9	12,2	12,1	12,3	12,4	12,3	12,7	12,7	12,7	12,9	12,9	12,8
	**75 years**	10,5	10,6	10,9	11,0	11,1	11,0	11,2	11,4	11,2	11,5	11,4	11,6	11,7	11,6	12,0	12,0	12,0	12,2	12,2	12,1
	**76 years**	9,9	10,0	10,3	10,4	10,5	10,3	10,5	10,7	10,5	10,9	10,8	10,9	11,0	10,9	11,3	11,3	11,3	11,5	11,5	11,4
	**77 years**	9,3	9,4	9,7	9,7	9,8	9,7	9,9	10,0	9,9	10,2	10,1	10,2	10,3	10,2	10,6	10,6	10,7	10,8	10,8	10,7
	**78 years**	8,8	8,8	9,1	9,1	9,2	9,1	9,2	9,4	9,3	9,6	9,5	9,6	9,7	9,6	10,0	9,9	10,0	10,1	10,2	10,1
	**79 years**	8,2	8,2	8,5	8,5	8,6	8,6	8,6	8,8	8,7	9,0	8,9	9,0	9,1	9,0	9,3	9,3	9,4	9,5	9,5	9,4
	**80 years**	7,6	7,6	8,0	8,0	8,1	8,0	8,1	8,3	8,1	8,4	8,3	8,4	8,5	8,4	8,7	8,7	8,8	8,9	8,9	8,8

	**81 years**	7,1	7,1	7,4	7,4	7,5	7,4	7,5	7,7	7,6	7,8	7,8	7,9	7,9	7,8	8,2	8,2	8,2	8,3	8,3	8,2
	**82 years**	6,7	6,6	6,9	6,9	7,0	6,9	7,0	7,2	7,1	7,3	7,2	7,3	7,4	7,3	7,6	7,6	7,6	7,7	7,8	7,6
	**83 years**	6,2	6,1	6,5	6,5	6,6	6,4	6,5	6,7	6,6	6,8	6,7	6,8	6,9	6,7	7,1	7,1	7,1	7,2	7,2	7,1
	**84 years**	5,8	5,7	6,0	6,0	6,1	6,0	6,0	6,3	6,1	6,3	6,3	6,4	6,4	6,3	6,6	6,6	6,6	6,7	6,7	6,6
	**85 years**	5,4	5,3	5,6	5,6	5,6	5,5	5,6	5,8	5,6	5,8	5,8	5,9	6,0	5,8	6,1	6,1	6,1	6,2	6,3	6,1
	**86 years**	5,0	5,0	5,3	5,2	5,2	5,2	5,2	5,4	5,2	5,4	5,4	5,5	5,5	5,4	5,7	5,7	5,6	5,8	5,8	5,7
	**87 years**	4,7	4,6	4,9	4,8	4,9	4,8	4,8	5,1	4,9	5,0	5,0	5,1	5,1	5,0	5,3	5,3	5,2	5,3	5,4	5,2
	**88 years**	4,3	4,3	4,5	4,4	4,5	4,4	4,5	4,7	4,5	4,7	4,6	4,7	4,7	4,6	4,9	4,9	4,9	4,9	5,0	4,8
	**89 years**	4,0	4,0	4,2	4,1	4,1	4,1	4,2	4,4	4,2	4,3	4,3	4,3	4,4	4,3	4,5	4,5	4,5	4,6	4,6	4,5
	**90 years**	3,7	3,6	3,9	3,8	3,8	3,8	3,8	4,1	3,9	4,0	4,0	4,0	4,0	3,9	4,2	4,2	4,2	4,3	4,3	4,2
	**91 years**	3,4	3,4	3,6	3,5	3,5	3,5	3,5	3,8	3,6	3,7	3,7	3,7	3,7	3,7	3,9	3,9	3,9	3,9	4,0	3,9
	**92 years**	3,1	3,1	3,4	3,2	3,2	3,2	3,2	3,6	3,4	3,5	3,4	3,5	3,5	3,4	3,6	3,6	3,6	3,7	3,7	3,6
	**93 years**	2,8	2,8	3,1	3,0	3,0	2,9	3,0	3,3	3,2	3,3	3,2	3,2	3,3	3,2	3,4	3,4	3,4	3,4	3,5	3,4
	**94 years**	2,6	2,6	2,8	2,7	2,7	2,7	2,7	3,1	3,0	3,0	3,0	3,0	3,1	3,0	3,2	3,2	3,2	3,2	3,3	3,1
	**95 years**	2,4	2,4	2,6	2,5	2,5	2,4	2,4	2,9	2,8	2,9	2,8	2,8	2,9	2,8	3,0	3,0	3,0	3,0	3,0	2,9
	**96 years**	2,1	2,1	2,3	2,2	2,2	2,2	2,2	2,7	2,6	2,7	2,6	2,7	2,7	2,6	2,8	2,8	2,8	2,8	2,8	2,8
	**97 years**	1,9	1,9	2,1	2,0	2,0	2,0	2,0	2,5	2,4	2,5	2,5	2,5	2,5	2,5	2,6	2,6	2,6	2,6	2,7	2,6
	**98 years**	1,7	1,7	1,9	1,8	1,8	1,8	1,8	2,4	2,3	2,3	2,3	2,3	2,3	2,3	2,4	2,4	2,4	2,5	2,5	2,4
	**99 years**	1,5	1,5	1,7	1,6	1,6	1,6	1,6	2,2	2,1	2,2	2,2	2,2	2,2	2,2	2,3	2,3	2,3	2,3	2,3	2,3
	**100 years**	1,4	1,4	1,5	1,4	1,4	1,4	1,4	2,1	2,0	2,1	2,0	2,0	2,1	2,0	2,1	2,1	2,1	2,2	2,2	2,1
